# mSphere of Influence: Revisiting the central dogma, again!

**DOI:** 10.1128/msphere.00398-24

**Published:** 2024-10-16

**Authors:** Parimal Samir

**Affiliations:** 1 Department of Microbiology and Immunology, The University of Texas Medical Branch, Galveston, Texas, USA; The University of Texas Medical Branch at Galveston, Galveston, Texas, USA

**Keywords:** defense-associated reverse transcriptase, phage defense, host–pathogen interactions

## Abstract

Dr. Parimal Samir works in the field of host–pathogen interactions. In this mSphere of Influence article, he reflects on how the manuscript entitled “*De novo* gene synthesis by an antiviral reverse transcriptase” by Samuel Sternberg and colleagues made an impact by reminding him that there is still so much to discover in life sciences.

## COMMENTARY

The idea that information flows from DNA to RNA to protein with an exception involving reverse transcription has stood the test of time. Protein-coding genes have been known to be encoded in a nucleic acid genome, which could be DNA or RNA. Some genes require chromosomal rearrangements and alternative splicing before they can be expressed into a protein, for example, B- and T-cell receptors. These processes can also expand the repertoire of proteins that can be expressed. But fundamentally, new genes are thought to arise only through duplications and mutations of existing genes. *De novo* synthesis of a protein-coding gene was considered impossible. Not anymore. In a recent study published in *Science*, researchers have uncovered evidence of a programmed *de novo* synthesis pathway for a protein-coding gene in bacteria (1). This process was found to confer resistance against bacteriophage infection. There are two reasons this article was chosen for my commentary. First, this study is a tour de force with multiple conceptual and methodological innovations. Second, it was also inspiring to read the scientific journey of the researchers as they struggled with technical challenges and came up with new solutions to move forward.

Samuel Sternberg and colleagues were studying the defense-associated reverse transcriptase (DRT) system present in many bacteria (2). DRTs are a type of mobile genetic element. Intriguingly, DRTs are associated with only one protein-coding open reading frame (ORF) that codes for a reverse transcriptase (RT). Previous studies had found that DRTs play a role in protecting the host against bacteriophage infections (3). Since DRTs contain only one ORF, this suggests that reverse transcription was necessary and sufficient for phage defense. Researchers developed an immunoprecipitation-based approach to identify an RNA product(s) of the DRT2 system from *Klebsiella pneumoniae* (kpnDRT2) and then the cDNA resulting from reverse transcription. They found that the DRT system codes for a large non-coding RNA (ncRNA) and a complementary cDNA. They then applied a fragment deletion approach to identify sequences in the ncRNA or cDNA responsible for phage defense. Surprisingly, all the fragments had lost phage defense capabilities. Not only that, but some of the mutants had also lost the ability to make the cDNA. Looking closely at the sequencing data, they found an even more surprising finding. In the WT kpnDRT2 system, a large majority of the reads that mapped to the cDNA had 3′ extensions that were identical to a sequence close to the 5′ end of the same cDNA. This indicated that the cDNA was in fact a concatemer with a precise head-to-tail junction (ccDNA). RNA circularization followed by rolling circle reverse transcription could be a mechanism by which ccDNA is generated. But the researchers did not find any evidence of RNA circularization. In fact, they found an even more astounding mechanism. The RT was jumping back toward the 5′ end of the ncRNA guided by RNA secondary structures. Although researchers still called this process rolling circle reverse transcription, it is mechanistically more of a recurrent programmed retrograde template jumping and continuing to reverse transcribe. Consistent with a role for the kpnDRT2 system in phage defense, the amount of ccDNA was increasing in cells infected with the *T5* phage. This process was also generating an ORF that did not have a stop codon, which the researchers called nearly endless ORF (*neo*). After overcoming several technical challenges, mainly due to the toxicity of the *neo* gene product, they showed that the polypeptide encoded by the *neo* gene was indeed responsible for phage defense. The expression of *neo* gene induced dormancy in infected cells, thus inhibiting phage replication and protecting the host.

To my knowledge, this is the first report of *de novo* protein-coding gene synthesis from a non-coding template. It remains to be seen how prevalent this process is in nature. If someone had asked me earlier whether such a process is possible, my answer would have been an emphatic no. Even while reading the paper, I kept wondering why nature even has to evolve in such a convoluted way to express a defense protein. And why is an RNA without a stop codon not being degraded. But in the end, it made complete sense. There is such a strong fitness cost of expressing the *neo* gene even at low levels, that conventional regulatory mechanisms of gene expression would not be sufficient. This study emphasized that there is so much more for us to discover and reminded me to stay humble, because I still know so little!

## References

[1] Tang S, Conte V, Zhang DJ, Žedaveinytė R, Lampe GD, Wiegand T, Tang LC, Wang M, Walker MWG, George JT, Berchowitz LE, Jovanovic M, Sternberg SH. 2024. De novo gene synthesis by an antiviral reverse transcriptase. Science 386:eadq0876. doi:10.1126/science.adq0876 39116258 PMC11758365

[2] González-Delgado A, Mestre MR, Martínez-Abarca F, Toro N. 2021. Prokaryotic reverse transcriptases: from retroelements to specialized defense systems. FEMS Microbiol Rev 45:fuab025. doi:10.1093/femsre/fuab025 33983378 PMC8632793

[3] Gao L, Altae-Tran H, Böhning F, Makarova KS, Segel M, Schmid-Burgk JL, Koob J, Wolf YI, Koonin EV, Zhang F. 2020. Diverse enzymatic activities mediate antiviral immunity in prokaryotes. Science 369:1077–1084. doi:10.1126/science.aba0372 32855333 PMC7985843

